# Food insecurity and frailty among women with and without HIV in the United States: a cross‐sectional analysis

**DOI:** 10.1002/jia2.25751

**Published:** 2021-06-14

**Authors:** Judy Y Tan, Lila A Sheira, Edward A Frongillo, Deborah Gustafson, Anjali Sharma, Daniel Merenstein, Mardge H Cohen, Elizabeth Golub, Andrew Edmonds, Igho Ofotokun, Margaret Fischl, Deborah Konkle‐Parker, Torsten Neilands, Phyllis Tien, Sheri D Weiser

**Affiliations:** ^1^ Center for AIDS Prevention Studies University of California San Francisco (UCSF) San Francisco CA USA; ^2^ Division of HIV, ID and Global Medicine University of California San Francisco (UCSF) San Francisco CA USA; ^3^ Department of Health Promotion, Education, and Behavior University of South Carolina Columbia SC USA; ^4^ Department of Neurology State University of New York Downstate Health Sciences University Brooklyn NY USA; ^5^ Department of Medicine Albert Einstein College of Medicine Bronx NY USA; ^6^ Department of Family Medicine Georgetown University Medical Center Washington DC USA; ^7^ Department of Medicine Stroger Hospital of Cook County Health Chicago IL USA; ^8^ WIHS Data Management Center Bloomberg School of Public Health Department of Epidemiology Johns Hopkins University Baltimore MD USA; ^9^ Department of Epidemiology Gillings School of Global Public Health University of North Carolina at Chapel Hill Chapel Hill NC USA; ^10^ School of Medicine Department of Medicine Emory University Atlanta GA USA; ^11^ Grady Healthcare System Atlanta GA USA; ^12^ Miller School of Medicine University of Miami Coral Gables FL USA; ^13^ Department of Medicine/Division of Infectious Diseases School of Nursing School of Population Health Sciences University of Mississippi Medical Center Jackson MS USA; ^14^ Department of Medicine San Francisco and Medical Service Department of Veteran Affairs Medical Center University of California San Francisco CA USA

**Keywords:** Food insecurity, frailty, HIV, women

## Abstract

**Introduction:**

Frailty is frequently observed among people with HIV, and food insecurity is associated with frailty in the general population. Evidence is scarce on the associations between food insecurity and frailty among women with HIV who may be particularly vulnerable to the impacts of food insecurity. The goal of this study was to assess associations between food insecurity and frailty among women with and without HIV.

**Methods:**

There were 1265 participants from the Women’s Interagency HIV Study who participated in frailty assessments in 2017. Frailty was measured using the Fried Frailty Phenotype, and women were subsequently categorized as robust, pre‐frail or frail. Food insecurity was assessed using the U.S. Household Food Security Survey Module, with women categorized as having high, marginal, low or very low food security. Multinomial logistic regression models were conducted to examine cross‐sectional associations between food insecurity and frailty while adjusting for socio‐demographic, behavioural and HIV status covariates.

**Results and discussion:**

Approximately one‐third (31.9%) of the women had marginal, low or very low food security, and the proportions of women who met the criteria for frailty or pre‐frailty were 55.6% and 12.4% respectively. In the adjusted model, the relative risk ratio (RRR) of frailty for women with very low food security versus women with high food security was 3.37 (95% CI [1.38 to 8.24], *p* < 0.01); the corresponding RRR of pre‐frailty was 3.63 (95% CI [1.76 to 7.51], *p* < 0.001). Higher annual household income was associated with lower RRRs of frailty or pre‐frailty (*p* < 0.01). Similarly, older age was associated with more frequent frailty (RRR=1.06, 95% CI [1.03 to 1.09], *p* < 0.001). HIV serostatus was not significantly associated with either pre‐frailty (RRR=0.97, 95% CI [0.71 to 1.31]) or frailty (RRR=0.75, 95% CI [0.48 to 1.16]).

**Conclusions:**

Very low food security was associated with more frequent frailty and pre‐frailty among women with and without for HIV. HIV serostatus was not associated with frailty.

## 1 INTRODUCTION

Food insecurity, or having limited access or ability to procure nutritional foods in socially acceptable ways [1], is associated with frailty, a multidimensional phenotype characterized by reduced strength, endurance and physiologic function [2]. Food insecurity and frailty are associated with lower daily energy intake, greater likelihood of obesity or being abnormal weight and poorer nutritional status overall [3, 4]. These characteristics are associated with greater risks for further health decline [5, 6, 7].

Frailty is prevalent among people with HIV, but there are limited data on food insecurity and frailty within the context of HIV [8, 9, 10, 11, 12, 13]. Frailty is commonly assessed using the Fried Frailty Phenotype criteria, composed of weight loss, exhaustion, physical inactivity, slow walking speed and weak grip strength. Individuals with three or more of these criteria are characterized as frail, those with one or two as pre‐frail and those with none as robust [2]. Many people with HIV experience health complications observed among the elderly in the general population but at an earlier age (i.e. 50 years) [14, 15]. The premature aging may be due to HIV disease itself, antiretroviral treatment toxicity or chronic inflammation, contributing to the phenomenon of accelerated aging [14, 15]. It is also possible that the high prevalence of premature age‐related conditions is due to non‐HIV‐related factors, such as social or behavioural factors [16]. As a key contributor to poor quality of life and functional status, frailty is associated with adverse outcomes among people with HIV, including fractures, falls and mortality [16, 17, 18, 19]. Frailty is also associated with lower CD4 count, lower nadir CD4 count and detectable viral load [20, 21]. Understanding modifiable risk factors for the development and progression of frailty is a priority for reducing morbidity and improving the quality of life of people with HIV.

Food insecurity may contribute to the high prevalence of frailty observed among people with HIV [3, 22, 23]. Frail persons with HIV are more likely to report food insecurity compared to those who are robust [22]. This finding was consistent across seven indicators of food insecurity (i.e. worrying about food running out, having only a few foods on hand, food not lasting, running out of food, affordability of food, going hungry and eating less) [22]. Among people with HIV, food insecurity is strongly associated with both falling and having balance problems in the past year, and evidence also suggests an association between food insecurity and strength and flexibility [24].

Women are disproportionally impacted by food insecurity due to gender‐based inequality in access to and control over resources [1, 25, 26, 27]. Relative to men with and without HIV, women have more frequent frailty, as well as other geriatric syndromes associated with frailty, such as falls and fracture [8, 10, 11]. Evidence in older populations without HIV strongly suggests that the relationship between frailty and mortality is greater among women than among men [28]. Research is scarce about factors associated with frailty and pre‐frailty among women as prior studies included cohorts that were predominantly men [29]. Moreover, the contribution of food insecurity to frailty over and above the impact of HIV infection remains unclear due to small sample sizes and a lack of a HIV‐negative comparison group in prior studies [24].

To address current research gaps, this study examined the association between food insecurity and frailty among women with and without HIV in the United States with multivariable adjustment for social‐behavioural factors suggested by the literature (smoking, substance use, alcohol use) [30, 31]. To our knowledge, this is the first study to examine the associations between food insecurity and frailty among demographically similar women with and without HIV.

## 2 METHODS

The Women’s Interagency HIV Study (WIHS, now known as the MACS/WIHS Combined Cohort [MWCCS] Study) is a multi‐centre, prospective cohort study in the United States established in 1994 that enrolled demographically similar women with and without HIV [32]. A cross‐sectional sample of 1265 participants from this cohort participated in frailty assessments between April and September of 2017 at nine WIHS sites: San Francisco/Bay Area, CA; The Bronx, NY; Brooklyn, NY; Washington, DC; Chicago, IL; Chapel Hill, NC; Birmingham, AL/Jackson, MS; Miami, FL; and Atlanta, GA. Details of cohort recruitment and demographics have been previously published; key demographic, behavioural and relevant clinical characteristics are comparable between the HIV‐seropositive and HIV‐seronegative samples [32, 33]. Participants provided written informed consent and were compensated for participation. This secondary study was approved by the institutional review board at each clinical research site’s institution and by the WIHS Executive Committee.

### 2.1 Independent variable

Participants completed a measure of food security using the U.S. Household Food Security Survey Module [34]. Participants were categorized as having high (no affirmations), marginal (one to two affirmations), low (three to five affirmations) and very low (six to ten affirmations) food security [34].

### 2.2 Dependent variable

Participants were assessed with a modified Fried Frailty Phenotype used in previous WIHS studies [10, 11, 18] that were composed of five components: unintentional weight loss, fatigue, low physical activity, measured weakness per grip strength and slow gait speed. Unintentional weight loss was assessed using a yes/no response to the item, “Since your last visit, have you had unintentional weight loss of at least 10 pounds?” Fatigue was assessed using a yes/no response to the item, “During the past four weeks, as a result of your physical health, have you had difficulty performing your work or other activities (for example, it took extra efforts)?” Low physical activity was assessed using a yes/no response to the item, “Does your health now limit you in vigorous activities, such as running, lifting heavy objects or participating in strenuous sports?” Grip strength was assessed using a hand‐held dynamometer with maximum force from the dominant hand. Gait speed was measured using a three to five metre timed gait. Slow gait and reduced grip strength were defined as the bottom quintile of performance; fatigue, weight loss and physical activity were based on self‐report [10, 35]. Additional details on score calculations are have been previously published [35]. Participants were categorized as robust (meeting none of the five criteria), pre‐frail (meeting one or two of the five criteria) or frail (meeting three or more of the five criteria).

### 2.3 Covariates

Covariates were selected based on prior research and a conceptual framework for understanding the impact of food insecurity on HIV outcomes [30, 31]. Covariates were HIV serostatus (negative [reference] vs. positive) as confirmed by a reactive HIV serology and confirmatory test, age in years (continuous), annual household income (<$12,000 [reference], $12,001‐$24,000, $24,001‐$36,000, $36,001‐$75,000, >$75,000), education (less than high school/high school equivalency vs. high school/high school equivalency [reference] or higher), cigarette smoking status (not current [reference] vs. current smoker), alcohol use (fewer than [reference] vs. more than or equal to seven drinks per week since the last visit) and substance use (none [reference] vs. any self‐reported use of cocaine, crack, heroin, methamphetamine, hallucinogens, club drugs, non‐prescribed narcotics or any other illicit recreational drugs, excluding any form of marijuana, in the last six months). Race/ethnicity was examined in the univariate analysis but not included in the multivariable analysis because it is not a covariate as suggested by the literature.

Chi‐square tests were used to examine differences in proportions, and the Kruskal–Wallis test was used to compare continuous variables. Multinomial logistic regression models were conducted to examine cross‐sectional associations between food security and frailty status (robust, pre‐frail and frail) after adjusting for HIV serostatus, age, years of education, annual household income, cigarette smoking status, substance use and alcohol use. Categorical covariates were included as indicator variables. Effect modification by HIV status was examined. Relative risk ratios (RRR) were computed by exponentiating the multinomial logistic regression coefficients [36]. The sample for the multinomial analysis was 1183 because 58 of the 1265 assessed for frailty had not been assessed for food security and 24 had missing data for income (n = 23) or employment (n = 1).

## 3 RESULTS AND DISCUSSION

Table 1 shows the distributions of food security and HIV serostatus, demographic factors and behavioural risk factors, by frailty status.

**Table 1:** Characteristics of women with and without HIV by frailty status, WHIS, 2017 (*N* = 1265)

	Overall Sample (*N* = 1265)	Robust (*N* = 405) (32.0%)	Pre‐frail (*N* = 703) (55.6%)	Frail (*N* = 157) (12.4%)	*p*‐value^a^
Food security					
High	823 (68.2%)	290 (74.7%)	442 (66.0%)	91 (61.1%)	<0.01
Marginal	166 (13.8%)	48 (12.4%)	94 (14.0%)	24 (16.1%)	
Low	131 (10.9%)	41 (10.6%)	71 (10.6%)	19 (12.8%)	
Very low	87 (7.2%)	9 (2.3%)	63 (9.4%)	15 (10.1%)	
Median age (IQR)	53.0 (47.0, 58.0)	51.0 (46.0, 57.0)	53.0 (47.0, 58.0)	55.0 (51.0, 59.0)	<0.01
HIV positive	920 (72.7%)	295 (72.8%)	522 (74.3%)	103 (65.6%)	0.09
Race/Ethnicity					
Non‐Hispanic White	113 (8.9%)	32 (7.9%)	68 (9.7%)	13 (8.3%)	0.75
Hispanic	181 (14.3%)	62 (15.3%)	93 (13.2%)	26 (16.6%)	
Non‐Hispanic African‐American/Black	923 (73.0%)	296 (73.1%)	517 (73.5%)	110 (70.1%)	
Other	48 (3.8%)	15 (3.7%)	25 (3.6%)	8 (5.1%)	
Annual household income					
<$12,000	609 (49.1%)	162 (40.7%)	340 (49.3%)	107 (69.9%)	<0.01
$12,001 to $24,000	257 (20.7%)	62 (15.6%)	166 (24.1%)	29 (19.0%)	
$24,001 to $36,000	145 (11.7%)	52 (13.1%)	89 (12.9%)	4 (2.6%)	
$36,001 to $75,000	154 (12.4%)	72 (18.1%)	71 (10.3%)	11 (7.2%)	
$75,001+	75 (6.0%)	50 (12.6%)	23 (3.3%)	2 (1.3%)	
High school education	413 (32.7%)	107 (26.4%)	236 (33.6%)	70 (44.6%)	<0.01
Current smoking	501 (39.6%)	128 (31.6%)	292 (41.5%)	81 (51.6%)	<0.01
Substance use^b^	114 (9.0%)	28 (6.9%)	60 (8.5%)	26 (16.6%)	<0.01
Alcohol use^c^	104 (8.2%)	27 (6.7%)	59 (8.4%)	18 (11.5%)	0.17

IQR, interquartile range; WIHS, The Women’s Interagency HIV Study.

^a^ Chi‐square tests were used to examine differences in proportions, and the Kruskal–Wallis test was used to compare continuous variables

^b^ substance use is defined as any self‐reported use of cocaine, crack, heroin, methamphetamine, hallucinogens, club drugs, non‐prescribed narcotics or any other illicit recreational drugs excluding any form of marijuana in the last six months or since the last study visit

^c^ fewer than vs. more than or equal to seven drinks per week since the last visit.

The median age of the sample was 53.0 years (interquartile range [IQR]: 47.0, 58.0). Approximately one‐third (31.9%) of participants reported marginal, low, or very low food security. Of the total sample, 157 (12.4%) participants were assessed as frail, 703 (55.6%) as pre‐frail and 405 (32%) as robust. Women with very low food security had more frequent pre‐frailty and frailty relative to those with high food security, consistent with research showing a higher prevalence of frailty components (e.g. problems with balance, falling, strength and being sedentary) with lower food security [22, 37]. In the adjusted model that examined being frail and pre‐frail relative to being robust, the RRR of frailty among women with very low food security compared to those with high food security was 3.37 (95% CI [1.38 to 8.24], *p* < 0.01) (Table 2).

**Table 2:** Characteristics associated with pre‐frail and frail status among women with and without HIV, multinomial logistic regression models, WIHS, 2017 (n = 1183)

	Unadjusted RRR (95% CI)		Adjusted RRR (95% CI)	
	Pre‐frail (vs. robust)	Frail (vs. robust)	Pre‐frail (vs. Robust)	Frail (vs. robust)
Food security (high FS *Ref*)				
Marginal FS	1.28 (0.88 to 1.87)	1.59 (0.93 to 2.74)	1.07 (0.72 to 1.59)	1.24 (0.69 to 2.21)
Low FS	1.14 (0.75 to 1.72)	1.48 (0.82 to 2.67)	0.88 (0.57 to 1.36)	0.91 (0.47 to 1.73)
Very low FS	4.59 (2.25 to 9.38)	5.31 (2.25 to 12.54)	3.63 (1.76 to 7.51)	3.37 (1.38 to 8.24)
HIV (no HIV ref)	1.08 (0.82 to 1.42)	0.71 (0.48 to 1.06)	0.97 (0.71 to 1.31)	0.75 (0.48 to 1.16)
Age	1.01 (1.00 to 1.03)	1.06 (1.03 to 1.09)	1.01 (1.00 to 1.03)	1.06 (1.03 to 1.09)
≥High school education (<HS *Ref*)	0.71 (0.54 to 0.93)	0.45 (0.30 to 0.66)	0.97 (0.72 to 1.31)	0.64 (0.42 to 0.99)
Annual household income (<$12,000 *Ref*)				
$12,001 to $24,000	1.28 (0.90 to 1.80)	0.71 (0.43 to 1.17)	1.39 (0.97 to 1.99)	0.88 (0.52 to 1.49)
$24,001 to $36,000	0.82 (0.55 to 1.20)	0.12 (0.04 to 0.33)	0.93 (0.61 to 1.40)	0.13 (0.04 to 0.45)
$36,001 to $75,000	0.47 (0.32 to 0.69)	0.23 (0.12 to 0.46)	0.52 (0.35 to 0.78)	0.31 (0.15 to 0.64)
$75,001+	0.22 (0.13 to 0.37)	0.06 (0.52 to 0.84)	0.29 (0.16 to 0.51)	0.12 (0.03 to 0.52)
Current smoking (not current *Ref*)	1.54 (1.19 to 1.99)	2.31 (1.58 to 3.36)	1.29 (0.96 to 1.72)	1.63 (1.06 to 2.51)
Substance use^a^ (none *Ref*)	1.26 (0.79 to 2.00)	2.67 (1.51 to 4.72)	0.93 (0.54 to 1.60)	1.61 (0.82 to 3.15)
Alcohol use^b^ (fewer *Ref*)	1.28 (0.80 to 2.06)	1.81 (0.97 to 3.39)	1.15 (0.69 to 1.92)	1.12 (0.54 to 2.33)

Covariates included HIV serostatus, annual household income, education, cigarette smoking status, alcohol use and substance use. CI, confidence interval; FS, food security; Ref, reference group; RRR, relative risk ratio; WIHS, The Women’s Interagency HIV Study.

^a^ Substance use is defined as any self‐reported use of cocaine, crack, heroin, methamphetamine, hallucinogens, club drugs, non‐prescribed narcotics, or any other illicit recreational drugs, excluding any form of marijuana, in the last six months or since the last study visit

^b^ fewer than vs. more than or equal to seven drinks per week since the last visit.

Similarly, the RRR of pre‐frailty among women with very low food security compared to those with high food security was 3.63 (95% CI [1.76 to 7.51], *p* < 0.001). The association of food insecurity and frailty status did not differ by HIV serostatus (interaction term not shown). The inclusion of a matched HIV‐seronegative comparison group adds to the existing literature by demonstrating that, when food insecurity and other relevant demographic and behavioural risk factors were considered, women with HIV were not more or less at risk for frailty or pre‐frailty compared to HIV‐seronegative women.

Higher income was associated with less frequent frailty and pre‐frailty. Older age, less education and smoking were associated with more frequent frailty only, consistent with past research [22, 37] and with findings from the Multicenter AIDS Cohort Study (MACS) with men [38]. Alcohol use and HIV serostatus were not associated with frailty when food security and other relevant demographic and behavioural risk factors were considered. Substance use was associated with frailty with about the same RRR as for smoking but with a wider confidence interval because of its lower prevalence.

Pre‐frailty, defined as the presence of one or two of the Fried Frailty Phenotype components, is considered an intermediary state in the transitional process from robustness to frailty [4, 33]. The detection of pre‐frailty may represent an opportunity for preventive interventions as it has been found to be an independent predictor of disease [39]. Given the higher likelihood of transitioning to a robust state from pre‐frail than from frail states [40, 41], our findings highlight food security as a modifiable social‐structural interventional target for mitigating the early and potentially reversible stages of frailty in people with HIV. For the first time ever, the population with HIV is aging. These findings provide timely insights into the associations among a prevalent social determinant of health, food insecurity and conditions commonly experienced prematurely by an aging population with HIV that is growing.

### 3.1 Implications for research

Longitudinal research is needed to investigate directionality and potential mediators of the association between food insecurity and frailty, including poor nutritional status and inflammation [29, 31, 42, 43]. Among women with HIV, food insecurity is associated with increases in inflammation when adjusting for social–behavioural factors, HIV virological control and body mass index [44]. While studies among predominantly male samples suggest HIV infection to be an independent predictor of frailty [45, 46], other research strongly suggests that chronic inflammation, rather than HIV serostatus, may better predict incident frailty among people with HIV, especially among women [29, 43]. Food insecurity may be more common among women than men, and future research is needed to elucidate the gender‐specific pathways that food insecurity may lead to frailty among people with and without HIV.

### 3.2 Limitations

Because the design was cross‐sectional, the causal direction between food insecurity and frailty cannot be determined. We would expect food insecurity to impact frailty and not the reverse, however, as food insecurity can be a precursor to a poor nutritional status which is a component of frailty. Yet, some people with frailty could have more difficulty accessing food than those who are robust, and this study was not designed to differentiate between these possibilities.

## 4 CONCLUSIONS

Very low food security was associated with more frequent frailty or pre‐frailty among women with and without HIV. HIV serostatus was not associated with frailty. Frailty prevention and intervention efforts should consider addressing food security among the growing population of people with HIV.

## Competing interests

The authors have declared no conflict of interest.

## Authors’ Contributions

SW, PT and LS designed research; SW, LS, EAF and JYT analysed and interpreted the data; JYT and SW wrote the paper; SW had primary responsibility for final content. DG, DM, MHC, EG, AE, IO, MF, DK‐P, AS, PT and SW contributed to the research design. All authors read and approved the final manuscript.

## Abbreviations

HIV, human immunodeficiency virus; WIHS, Women’s Interagency HIV Study.
